# Mixed infection of ITPase-encoding potyvirid and secovirid in *Mercurialis perennis*: evidences for a convergent euphorbia-specific viral counterstrike

**DOI:** 10.1186/s12985-023-02257-y

**Published:** 2024-01-04

**Authors:** Mathieu Mahillon, Justine Brodard, Nathalie Dubuis, Paul Gugerli, Arnaud G. Blouin, Olivier Schumpp

**Affiliations:** Research Group Virology, Bacteriology and Phytoplasmology, Plant Protection Department, Agroscope, Nyon, Switzerland

**Keywords:** Comovirinae, Deltapartitivirus, Euphorbiaceae, Inosine, HGT, Mercurialis, Partitiviridae, Potyviridae, Potyvirus, Secoviridae

## Abstract

**Background:**

In cellular organisms, inosine triphosphate pyrophosphatases (ITPases) prevent the incorporation of mutagenic deaminated purines into nucleic acids. These enzymes have also been detected in the genomes of several plant RNA viruses infecting two euphorbia species. In particular, two ipomoviruses produce replicase-associated ITPases to cope with high concentration of non-canonical nucleotides found in cassava tissues.

**Method:**

Using high-throughput RNA sequencing on the wild euphorbia species *Mercurialis perennis*, two new members of the families *Potyviridae* and *Secoviridae* were identified. Both viruses encode for a putative ITPase, and were found in mixed infection with a new partitivirid. Following biological and genomic characterization of these viruses, the origin and function of the phytoviral ITPases were investigated.

**Results:**

While the potyvirid was shown to be pathogenic, the secovirid and partitivirid could not be transmitted. The secovirid was found belonging to a proposed new *Comovirinae* genus tentatively named "Mercomovirus", which also accommodates other viruses identified through transcriptome mining, and for which an asymptomatic pollen-associated lifestyle is suspected. Homology and phylogenetic analyses inferred that the ITPases encoded by the potyvirid and secovirid were likely acquired through independent horizontal gene transfer events, forming lineages distinct from the enzymes found in cassava ipomoviruses. Possible origins from cellular organisms are discussed for these proteins. In parallel, the endogenous ITPase of *M. perennis* was predicted to encode for a C-terminal nuclear localization signal, which appears to be conserved among the ITPases of euphorbias but absent in other plant families. This subcellular localization is in line with the idea that nucleic acids remain protected in the nucleus, while deaminated nucleotides accumulate in the cytoplasm where they act as antiviral molecules.

**Conclusion:**

Three new RNA viruses infecting *M. perennis* are described, two of which encoding for ITPases. These enzymes have distinct origins, and are likely required by viruses to circumvent high level of cytoplasmic non-canonical nucleotides. This putative plant defense mechanism has emerged early in the evolution of euphorbias, and seems to specifically target certain groups of RNA viruses infecting perennial hosts.

**Graphical Abstract:**

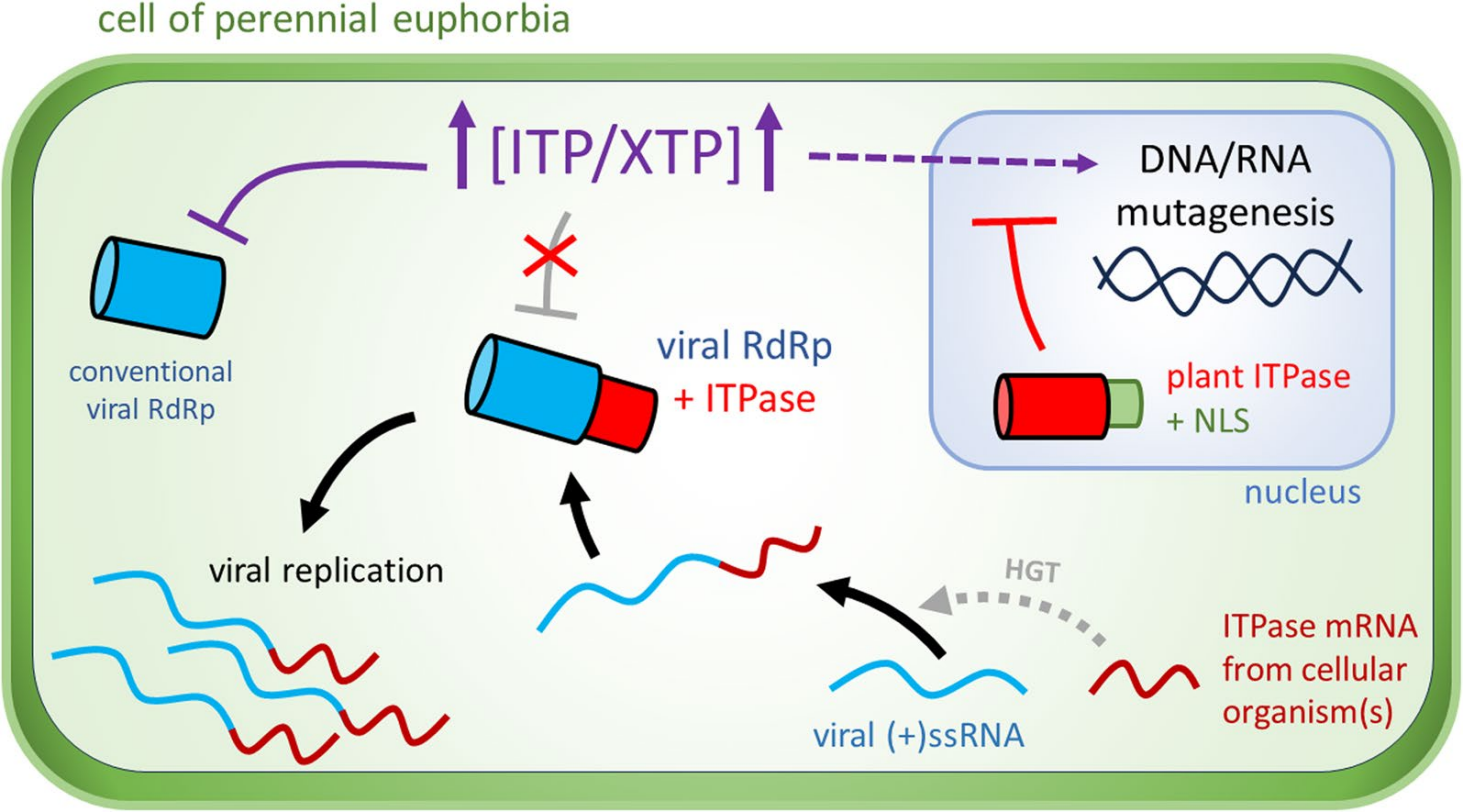

**Supplementary Information:**

The online version contains supplementary material available at 10.1186/s12985-023-02257-y.

## Background

The known plant virome is dominated by RNA viruses, in particular with positive-sense, single-stranded RNA [( +)ssRNA] genomes [26]. These viruses belong to *Riboviria*, a vast realm formally recognized in 2018 by the International Committee on Taxonomy of Viruses (ICTV). Plant ( +)ssRNA viruses possess relatively compact genomes that undergo rapid evolution due to high mutation rates [10, 52]. In parallel, they can also evolve by acquiring novel genes from cellular organisms or from other viruses via horizontal gene transfer (HGT) [9]. However, the capacity of plant ( +)ssRNA viral genomes to tolerate insertion is often limited, resulting in the rapid loss of foreign genes that penalize the virus fitness [70]. Nevertheless, there are instances where HGT leads to a stable insertion, and the transferred gene becomes an integral part of the genome. One notable example of such successful HGT involves *AlkB* that is spread in diverse clades of phytoviruses and which hypothetically repairs deleterious methylation damages [66]. Other genes acquired via HGT include genes coding for homologs of the heat shock protein [53], thaumatin [2, 32] and inosine triphosphate pyrophosphatase (ITPase). Notably, ITPases have been found in several ( +)ssRNA viruses and seem to represent a host-specific adaptation.

ITPases catalyze the hydrolysis of non-canonical nucleosides triphosphate (NTPs), mainly inosine and xanthosine triphosphate (ITP and XTP, respectively), which are produced by deamination of purines after oxidative stress for instance. In cellular organisms, these enzymes are therefore essential for preventing the incorporation of ITP and XTP into DNA and RNA, thereby avoiding their deleterious mutagenic effects [31, 37, 54, 60]. When it comes to phytoviruses, ITPases were first detected in two ipomoviruses, namely cassava brown streak virus (CBSV) and Ugandan cassava brown streak virus (UCBSV). Both viruses infect cassava (*Manihot esculenta*), an euphorbiaceous species that is an essential staple food in tropical and subtropical regions [36, 62]. The activities of CBSV and UCBSV ITPases have been demonstrated in vitro, and these enzymes were found to be associated with the viral replicases, protecting them from the high levels of ITP and XTP detected in cassava tissues [63, 65]. Indeed, it is believed that ITP/XTP act as nucleoside analogues that inhibit the viral replicases [65]. More recently, additional ITPases have been evidenced in the secovirid cassava torrado-like virus (CTLV) [21, 27], as well as in the potyvirus Euphorbia ringspot virus (EuRSV) which infects *Euphorbia milii* [3, 23, 35].

High levels of ITP/XTP accumulate in cassava, and the presence of an ITPase in EuRSV suggests that a similar built-up of non-canonical NTPs is likely to occur in *E. milii*, and potentially in other members of the *Euphorbiaceae* as well. This dicot family encompasses various species of flowering plants, including herbs, shrubs, trees, succulents and aquatic plants. Besides cassava, several other euphorbias hold economic significance, such as the rubber tree (*Hevea brasiliensis*), castor bean (*Ricinus communis*), and Indian gooseberry (*Phyllanthus emblica*). In Europe, *Mercurialis perennis*, commonly known as the "dog's mercury", is a wild euphorbia found in shaded woodland areas with minimal disturbance [20]. This perennial herb, toxic to humans and cattle [51], has not yet been screened for viruses. Here, two ( +)ssRNA and one double-stranded RNA (dsRNA) viruses infecting this species were characterized, leading to the identification of two novel virally-encoded ITPases for which the role and origin are discussed.

## Method

### Host range analysis

Symptomatic leaves of *M. perennis* were ground in a mortar with a pestle in cold phosphate buffer (20 mM Na_2_HPO_4_, pH 7.6, supplemented with 1 mM diethyldithiocarbamate). The obtained sap was used for mechanical inoculation using carborundum (400-mesh silicon carbide) as abrasive. At least four plants were inoculated for each tested species. Plants were then maintained for one month in greenhouse (20–25 °C, 14/10 h light/darkness) with daily monitoring.

### Virions semi-purification

For the semi-purification of potyviral particles, a method dedicated for Potato virus Y (PVY) virion was followed [16]. Briefly, 5 g of symptomatic leaves of *M. perennis* were first ground in 20 ml of cold extraction buffer (17 mM citric acid, 167 mM Na_2_HPO_4_, 10 mM ethylenediaminetetraacetic acid (EDTA), 1 M urea, 0.2% v/v thioglycolic acid, pH 7.0) supplemented with 1% (v/v) Triton, and the suspension was stirred for 30 min on ice. After a clarification step (10 min at 10,000 rpm), the supernatant was collected and placed on top of a 20% (w/v) sucrose cushion diluted in extraction buffer, which was followed by centrifugation for 2 h at 38,000 rpm. The obtained pellet was resuspended in extraction buffer without EDTA. The following day, one volume of chloroform:isoamyl alcohol (24:1) was added, and the resulting mix was carefully stirred on ice for 20 min. After a clarification step (20 min at 4,000 rpm), the supernatant was collected and placed on top of a 20% (w/v) sucrose cushion diluted in cold borate buffer (100 mM B_4_Na_2_O_7_.10H_2_O, 10 mM EDTA, pH 7.5), followed by centrifugation for 2 h at 38,000 rpm. The pellet was resuspended in 50 µl of borate buffer. Particles were then observed by transmission electron microscopy (TEM) with the Tecnai G2 electron microscope, as previously described [33].

### RNA isolation and illumina sequencing

Total RNA was extracted from leaves using a 3% cetyltrimethylammonium bromide (CTAB) method as previously described [34]. The extracted RNA was then treated with DNase Q1 (Qiagen) and sent to Fasteris (Switzerland) for high-throughput sequencing (HTS). After a ribodepletion step, a cDNA library of paired-ended, 150-bp reads was built and sequenced on an Illumina HiSeq 3000 platform. The HTS produced 45.6 M reads, of which 94% were of good quality (> Q30). The reads were trimmed with Trimmomatic [4] and then used for de novo assembly with Spades [45]. The obtained viral contigs (> 500 bp) were sorted based on BlastX searches in the NCBI “nr” database limited to the “virus” taxid. Reads coverages were determined using Bowtie2 [25] and Samtools [8]. The viral termini were obtained by Sanger sequencing, as follows: the 5’-termini were amplified using the SMARTer RACE 5’/3’ kit (Takara), and the 3’-termini were amplified using an oligo-dT in combination with specific viral primers (Additional file 1: Table S1), as previously described [33].

### Bioinformatics analyses

Viral genomes were analyzed on UGene [42] and Jalview [69]. Homologs for the viral proteins were identified through BlastP searches in the NCBI database. Conserved motifs and domains were detected using the online tool MOTIF Search (https://www.genome.jp/tools/motif/) including the Pfam and NCBI-CDD databases. The palmID tool of Serratus [12] was used to search for closely-related viral RNA-dependent RNA polymerase (RdRp) in the NCBI Sequence Read Archives (SRA) database. Matrices of pairwise amino acid (aa) identity were generated with the SDT software [38].

For phylogenetic analyses of the viruses, proteins from representative members of viral groups were first retrieved from NCBI. Alignments were then performed with MUSCLE [11]. The best substitution models were selected with ModelFinder [22] and used to build maximum-likelihood (ML) phylogenetic trees on IQ-tree [39] in combination with 1,000 replicates of ultrafast bootstrap [18]. The resulting ML trees were then curated on ITol [28].

For the global phylogenetic analysis of ITPases, homolog proteins were first retrieved from the NCBI Genbank database and grouped according to taxa. For each groups, the numbers of sequences was first reduced using CD-hit [29]. Clustal Omega [57] was then chosen to align these sequences with the ITPases encoded by all phytoviral isolates. After manual curation, the redundancy was reduced with Seqkit [55]. The final alignment combining 804 sequences with 646 columns was then used to build a ML phylogenetic tree as described above.

### Nucleic acid detection

Reverse transcription followed by polymerase chain reaction (RT-PCR) analyses were conducted on CTAB-extracted nucleic acids using the AMV reverse transcriptase and Taq polymerase (Promega), according to the manufacturer's instructions. Primers are listed in Additional file 1: Table S1. Thermocycler conditions were as follows: 48 °C for 45 min, 95 °C for 2 min followed by 30 cycles of 94 °C for 30 s, 60 °C for 30 s, 72 °C for 30 s, and a final elongation step at 72 °C for 5 min. Amplicons were visualized by agarose gel electrophoresis.

### ELISA

Leaf samples from plants located in Givrins were used to produce rabbit polyclonal antibodies targeting the potyvirid CP, which were subsequently used for DAS-ELISA as previously-described [46]. Absorbances were measured on a SpectraMax iD5 (Molecular Devices) after 1 h of incubation.

### Aphid transmission

Specimens of *Myzus persicae* and *Aphis fabae* were used for transmission assays as previously described [34]. Alate specimens were first placed on infected leaves of *M. annua* for 24 h and then carefully collected in a Petri dish. Insects were then placed onto two-week old healthy *M. annua* plants covered with insect-proof nets (Andermatt). After six days, the nets were removed and plants were sprayed with an insecticide (Gazelle® SG, Stähler). Plants were then maintained for one month in greenhouse conditions and then tested by RT-PCR.

## Results

### A triple viral infection in *Mercurialis perennis*

Symptoms of light green mosaic, vein yellowing and deformations were first observed in 1982 on the leaves of plants of *M. perennis* located along two walking paths in the forests bordering Givrins and Saint-Cergue (canton of Vaud, Switzerland). TEM analyses of these symptomatic plants highlighted the presence of particles associated with a putative potyvirid. Polyclonal antibodies raised against the particles efficiently recognized symptomatic samples from both locations, and showed cross reactivity with samples infected with the potyviruses turnip mosaic virus, plum pox virus and PVY-NTN (data not shown).

**Fig. 1:**
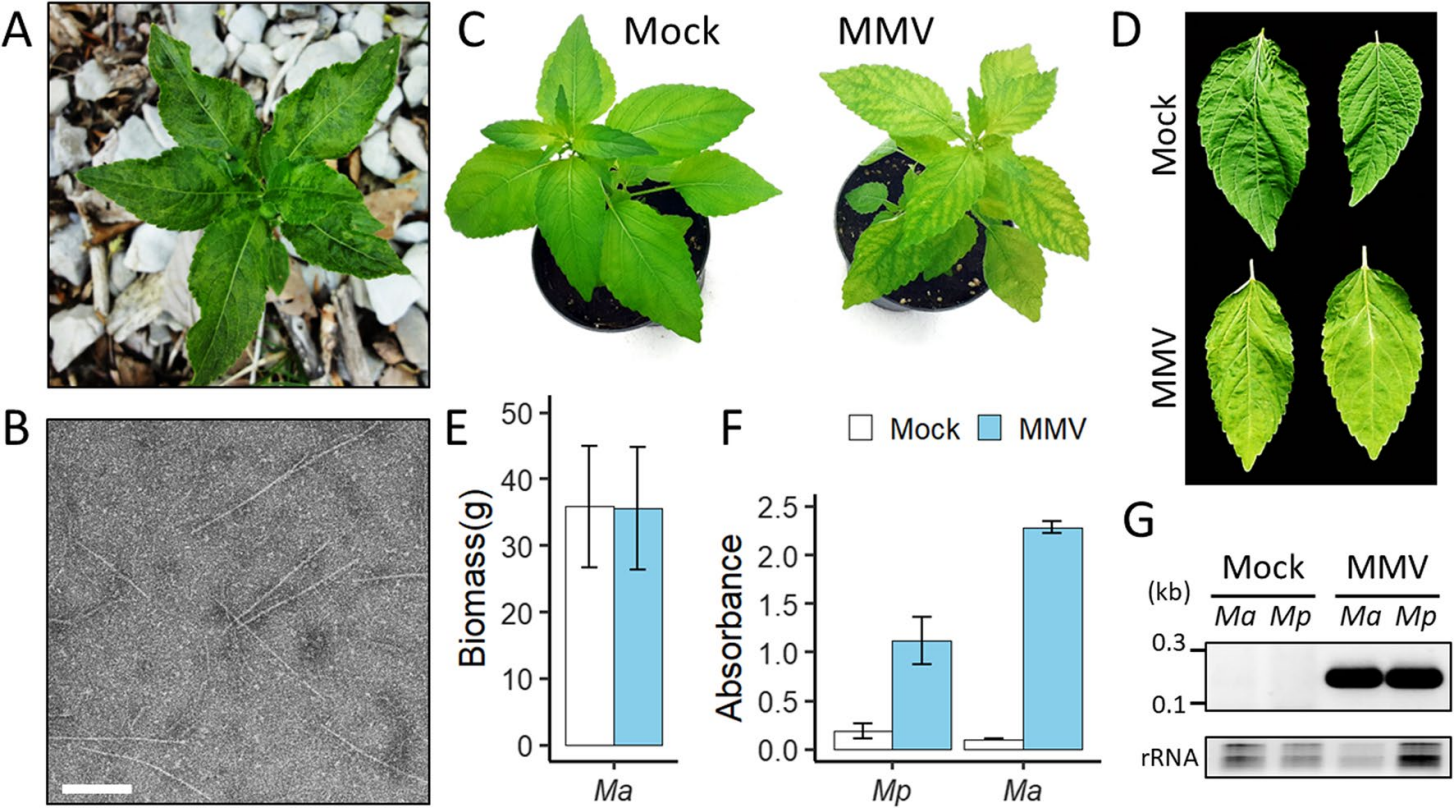
Symptoms, particles and pathogenicity of MMV. **A**. Symptoms on leaves of a plant of *M. perennis* (*Mp*) located at Saint-Cergue (Vaud, Switzerland). **B**. Electron micrograph of semi-purified flexuous virions. The white bar represents 500 nm. **C**. Comparison of mock (left) or MMV-infected (right) plants of *M. annua* (*Ma*), at 15 dpi. **D**. Comparison of mock (up) or MMV-infected (down) upper leaves of *Ma* at 30 dpi. **E**. Fresh aerial biomass (in gram) for mock (n = 8) or MMV-inoculated (n = 25) *Ma* plants at 35 dpi. Error bars represent standard deviations. **F**. Absorbance (405 nm) for DAS-ELISA analyses targeting MMV CP in leaf samples collected from *Mp* at 15 dpi, and *Ma* at 30 dpi. Error bars represent standard deviations (n = 6). **G**. Gel electrophoresis for the RT-PCR detection of MMV RNA in mock or MMV-infected plants. Plant ribosomal RNA (rRNA) are shown as loading control

In 2016, plants of *M. perennis* located at Saint-Cergue and exhibiting similar symptoms (Fig. 1A) were uprooted and have been maintained since then in greenhouses at Agroscope Changins (Nyon, Switzerland). Following a virion semi-purification protocol, flexuous particles *c.* 600–800 nm in length were clearly visible by TEM (Fig. 1B), suggesting that the potyvirid was still present in the wild population of *M. perennis*. The presence of a member of the genus *Potyvirus* was then confirmed by RT-PCR analysis using the generic primers pair CIFor/Rev [17] (data not shown).

HTS of total RNA extracted from the symptomatic leaves led to the assembly of five contigs (Additional file 1: Fig. S1) that were predicted to encode for proteins showing strong homology to known phytoviral proteins (BlastX hits E-value = 0, Additional file 1: Table S2). The largest viral contig (> 10 kb) corresponded to the genome of the potyvirus. Since this virus was proven to be pathogenic (see next section), the name “Mercurialis mosaic virus” (MMV) was hereafter used to describe it. Following Sanger sequencing of the termini, the full-length sequence of MMV has been deposited on NCBI under accession number OR544053. The second and third longest viral contigs (*c.* 6.0 and 3.5 kb, respectively) exhibited a bipartite genomic architecture similar to some members of the family *Secoviridae*, in particular to fabaviruses and comoviruses. These two contigs were therefore assigned to the RNA1 and 2 of a virus tentatively named “Mercurialis secovirus 1” (MSV1), for which the full-length sequences can be found under accession numbers OR544055-6, respectively. Lastly, the two smallest viral contigs (*c.*1.6 and 1.3 kb) corresponded to the RNAs of a partitivirus. The name “Mercurialis partitivirus 1” (MPV1) was hereafter for this virus, and the full-length sequence of its RNA1 and partial sequence of RNA2 are found under the accession numbers OR555822-3, respectively.

### Biological characterization

The presence of MMV, MSV1 and MPV1 was confirmed in the symptomatic leaves of *M. perennis* by RT-PCR targeting individual genomic segments. In order to further characterize these viruses, infected leaves were ground in a phosphate buffer, and the resulting sap was mechanically rubbed onto the leaves of several plant species. Inoculated plants of *M. perennis* reproduced the systemic mosaic symptoms 8–10 days after inoculation (dpi). Plants from the related herbaceous species *M. annua* developed interveinal chlorosis at 7 dpi (Fig. 1C). Upper leaves of this species eventually turned completely light green (Fig. 1D). On this species, while all the leaves of inoculated plants exhibited symptoms, there was no significant difference in terms of fresh aerial biomass in comparison to healthy plants (Fig. 1E). In both inoculated *Mercurialis* species, the presence of MMV was confirmed by DAS-ELISA and RT-PCR analyses (Fig. 1F, G). In contrast, there was no detection of the RNAs of MSV1 and MPV1 by RT-PCR (data not shown). Inoculations of *Nicotiana benthamiana*, *N. tabacum*, *N. occidentalis*, *N. clevelandii*, *Solanum lycopersicum*, *Chenopodium quinoa* and *C. amaranticolor* were unsuccessful for all three viruses as evidenced by the lack of symptom and negative RT-PCR results (data not shown). Inoculation of MMV was also tested on two other euphorbias, namely the poinsettia (*E. pulcherrima*) and marsh spurge (*E. palustris*), but did not lead to infection (data not shown).

Natural transmission of potyviruses is mediated by aphids in a non-persistent manner [15]. In order to test this trait for MMV, transmission assays were conducted on infected leaves of *M. annua* using specimens of *M. persicae* (n = 8) and *A. fabae* (n = 9). None of the recipient plants showed symptoms nor tested positive by RT-PCR, advocating that these aphid species are not efficient vectors. It should be noted that there was no sign of living aphids after the transmission period on the recipient plants, which indicate that these species may not be adapted to *M. annua*.

### Viral genomes analysis

**Fig. 2:**
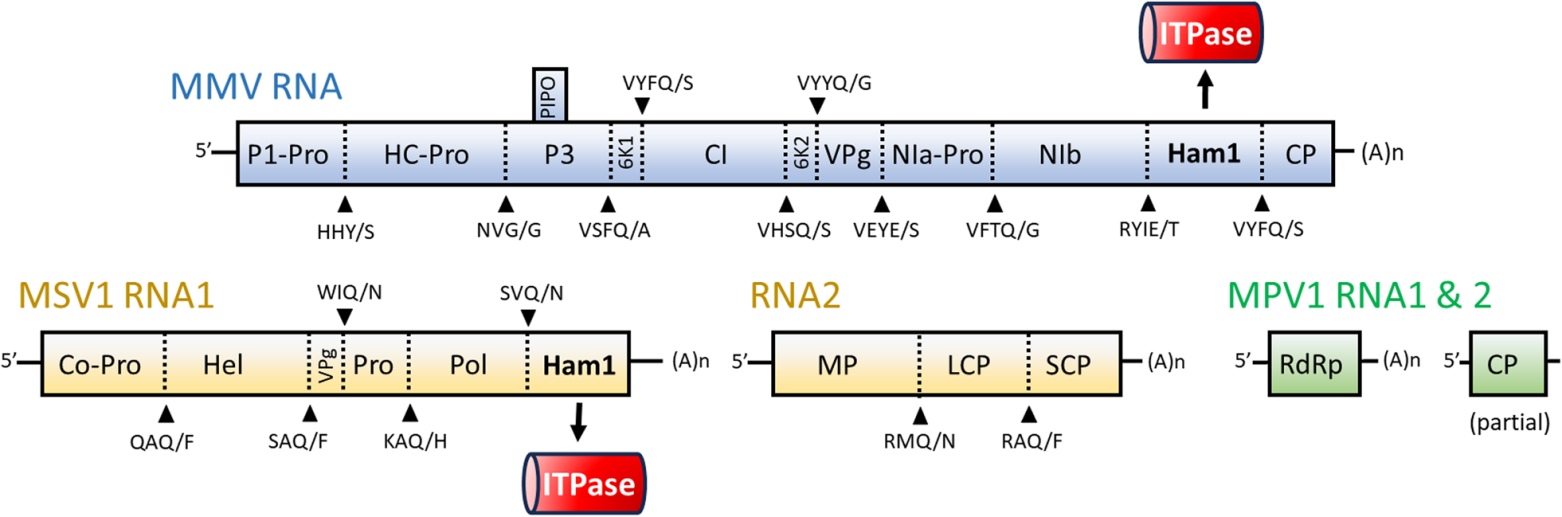
Schematic representation of the genomic RNAs of MMV (blue), MSV1 (orange) and MPV1 (green). Colored boxes represent ORFs (see main text and Additional file 1: Table S3 for details). Black arrows indicate the motifs associated with the potential cleavage sites (dotted lines). The phytoviral ITPases encoded by the Ham1 peptides are highlighted with a red background. A(n): poly(A) tail

A schematic representation of the three viral genomes is given in Fig. 2, and a detailed annotation of their open reading frames (ORFs) is provided in Additional file 1: Table S3.

The MMV RNA ends with a poly(A) tail and is predicted to produce a large protein of 3,309 aa. With the exception of bipartite bymoviruses, all potyvirids share a monopartite genome encoding a large polyprotein that is cleaved by three virally-encoded proteases (*i.e.* P1-Pro, HC-Pro and Nla-Pro) [44]. Putative cleavage sites can be identified on MMV polyprotein based on previous studies [1, 23]. Eleven individual peptides are therefore predicted, ten of which representing the conserved core modules of potyviral genomes [44]. Interestingly, an unusual peptide exhibiting an ITPase-associated Ham1 domain is found between the replicase (Nlb) and CP peptides (Fig. 2, red box in upper panel). In addition to the polyprotein, a small ORF encoding for 92 aa is predicted inside the P3 ORF, and it corresponds to the *Pretty interesting* *Potyviridae* ORF (PIPO) as evidenced by homology with other PIPO sequences (data not shown) and the presence of the preceding typical slippery ^3078^GAAAAAA motif [43].

Both RNAs of MSV1 end with a poly(A) tail and harbor a single ORF. Secovirids produce polyproteins that are cleaved by a virally-encoded 3C protease (Pro), for which the substrate-binding pocket (SBP) is ^1146^GVH in the case of the polyprotein of MSV1 RNA1 (1,981 aa). This SBP motif suggests that cleavages are likely to occur after Q residues [61]. Five potential cleavage sites are present in the polyprotein of MSV1 RNA1, producing five peptides also found in other secovirids and involved in replication and proteolysis. In addition, a sixth peptide harboring a Ham1 domain is found in C-terminal (C-ter) of the RdRp (Fig. 2, red box in lower panel). The polyprotein encoded by MSV1 RNA2 (990 aa) contains the three predicted peptides associated with *in planta* movement and virions.

The architecture of MPV1 bi-segmented genome is typical for a partitivirus, with both RNA encoding for a single ORF. The deduced protein sequence of these ORFs correspond to an RdRp (479 aa) and a putative CP (372 aa, partial), respectively.

### Phylogenetic and taxonomic analyses

**Fig. 3:**
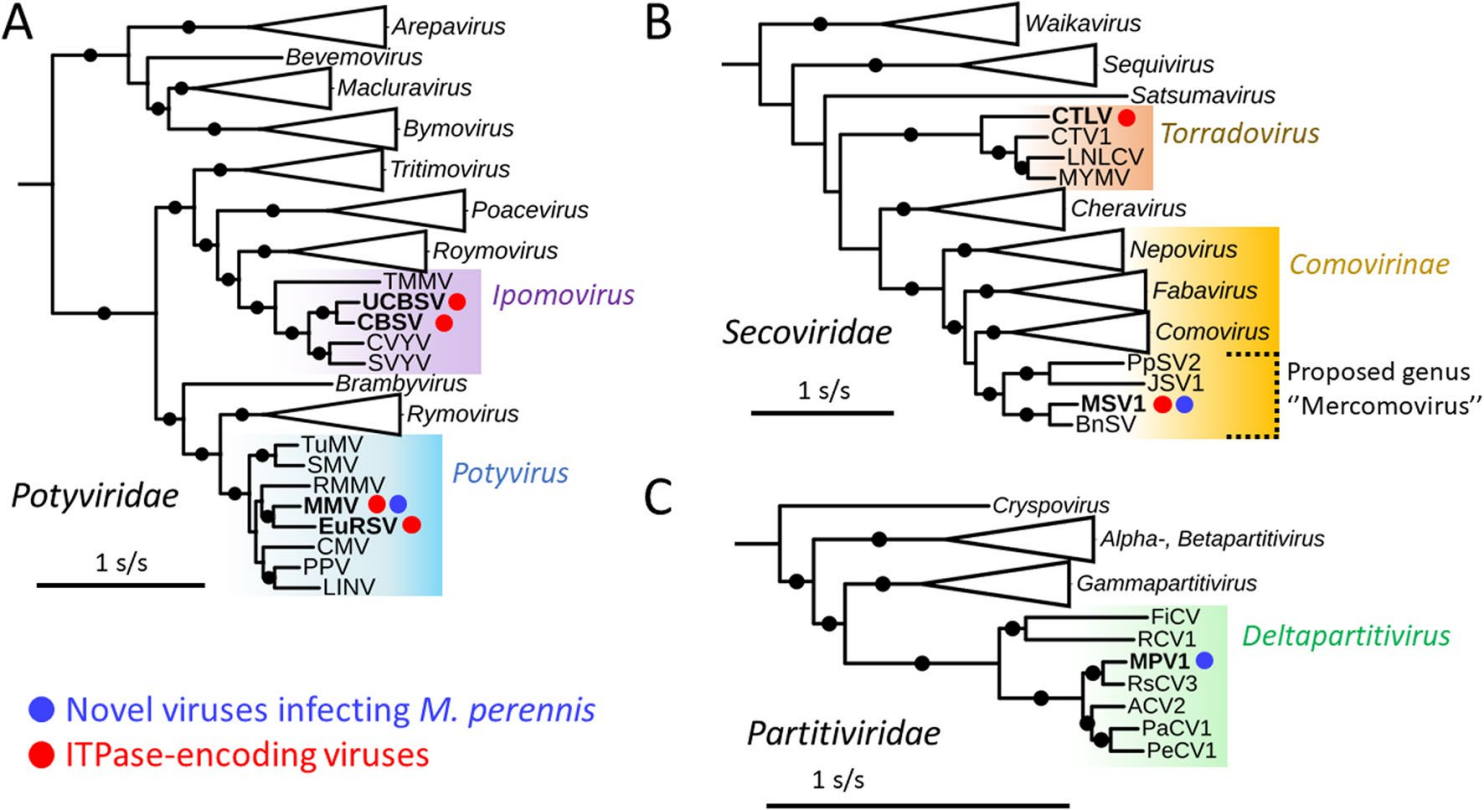
ML phylogenetic trees for MMV (**A**), MSV1 (**B**) and MPV1 (**C**) and their related viruses. The trees A and B were built using the substitution model LG + I + G4, and the model Blosum62 + F + I + G4 was used for the tree C. Black circles on branches indicate > 75% bootstrap support (1000 replicates). The scales are given in substitution by site. Accession numbers and full virus names are listed in Additional file 1: Table S4, S5 and S6. Members of the genera *Celavirus*, *Dicistrovirus* and *Picobirnavirus* were used to root the initial trees, respectively

*Potyviridae*, the largest family of plant ( +)ssRNA viruses, accommodates 12 genera [71]. A ML phylogenetic tree based on the Nlb peptide unambiguously places MMV within the genus *Potyvirus*, close to EuRSV (Fig. 3A). The polyproteins of MMV and EuRSV share only 53.4% identity, which is well below the species demarcation threshold (*i.e*. 82%) established by the ICTV [71], confirming that MMV represents a novel potyviral species.

The family *Secoviridae* comprises nine genera of monopartite or bipartite ( +)ssRNA viruses [14]. In a ML tree based on an alignment of the Pro-Pol regions (i.e. delimited by the conserved motifs GC and GDD, Fig. 3B), MSV1 is placed in a strongly-supported monophyletic clade within the subfamily *Comovirinae*. This clade also accommodates two viruses that were recently identified through transcriptome mining [56], namely “Boehmeria nivea secovirus” (BnSV) and “Paris polyphylla secovirus 2” (PpSV2). A third member has been recently identified in Jujube and named “jujube-associated secovirus 1” (JSV1) [72]. In the ML tree, MSV1 appears to be most closely related to BnSV, but these viruses only share 66.81% aa identity for the Pro-Pol region and should be thus considered as distinct species according to the ICTV criteria [14]. The MSV1-associated clade appears to be closer to the genus *Comovirus* than the other genera, although this topology is only supported by 67% of bootstrap replicates. In a ML tree built for the CP, MSV1 and related viruses form again a monophyletic group distinct from the other *Comovirinae* genera (Additional file 1: Fig. S2). In that case, the most closely related sequences correspond to members of the genus *Fabavirus*. Altogether, the distinct monophyletic lineages formed by the Pol-Pro and CP fulfill the conditions for the creation of a novel *Comovirinae* genus [14], for which the proposed name “Mercomovirus” derives from “Mercurialis comovirinae virus”.

The family *Partitiviridae* is a large group of dsRNA viruses infecting plants, fungi, chromists, oomycetes and arthropods [7, 64]. The ICTV currently recognizes five genera of partitiviruses, all named with a Greek prefix (*Alpha-*, *Beta-*, *Delta-* and *Gammapartitivirus*) except for the genus *Cryspovirus*. In a ML tree based on the RdRp, MPV1 clearly associates with members of *Deltapartitivirus* (Fig. 3C). The most closely related member is Raphanus sativus cryptic virus 3 (RsCV3), for which the proteins share 81.09% and 54.52% identity with MPV1 RdRp and CP, respectively, confirming that these viruses represent distinct species [64].

### Additional “mercomoviruses” in SRA datasets

**Fig. 4:**
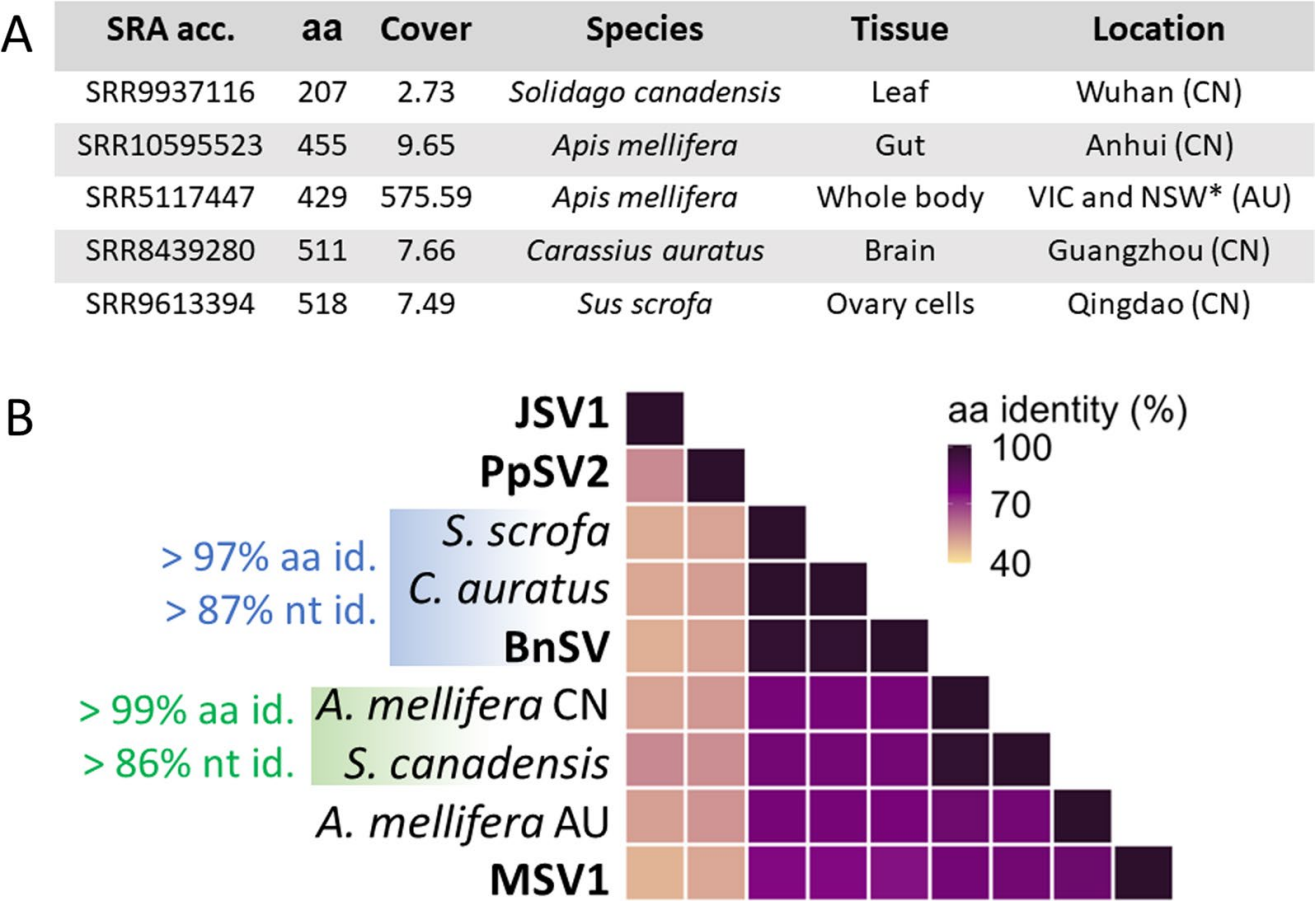
Identification of MSV1-related RdRp in SRA repositories.** A**. Description of the SRA-associated samples in which the mercomoviral RdRps are identified. AU: Australia; CN: China. *Victoria and New South Wales. **B**. Matrix of pairwise aa identities for the mercomoviral RdRps

A screening through SRA depositories using the Serratus palmID tool uncovered five partial RdRps representing additional “mercomoviral” sequences (Fig. 4A). Four of these sequences are associated with studies performed in China, corresponding to the transcriptomes of three animals and one plant [30, 67, 68, 73]. The last RdRp is derived from a bee metaviromic study conducted in Australia [49]. The read coverages of these sequences are quite variable,while the RdRp associated with Australian bees was reconstructed with high coverage, the sequencing depth obtained for the Chinese sequences was rather low (< 10x). A comparison of pairwise aa identities for these SRA-associated RdRps suggest that they represent at least three distinct species (Fig. 4B), which appear more similar to MSV1 and BnSV than PpSV2 and JSV1. In fact, the sequences found in two of the animal transcriptomes in China correspond to isolates of BnSV as indicated by the high nucleotides and aa identities. Interestingly, Blastn searches in these transcriptomes revealed the presence of numerous reads matching the plant-specific *rbcL* gene of *Boehmeria nivea* (Genbank MN189944.1, positions 55,954–57,402), suggesting that reads from BnSV-infected plant material may have contaminated these datasets.

### Phytoviral ITPase function and origins

**Fig. 5:**
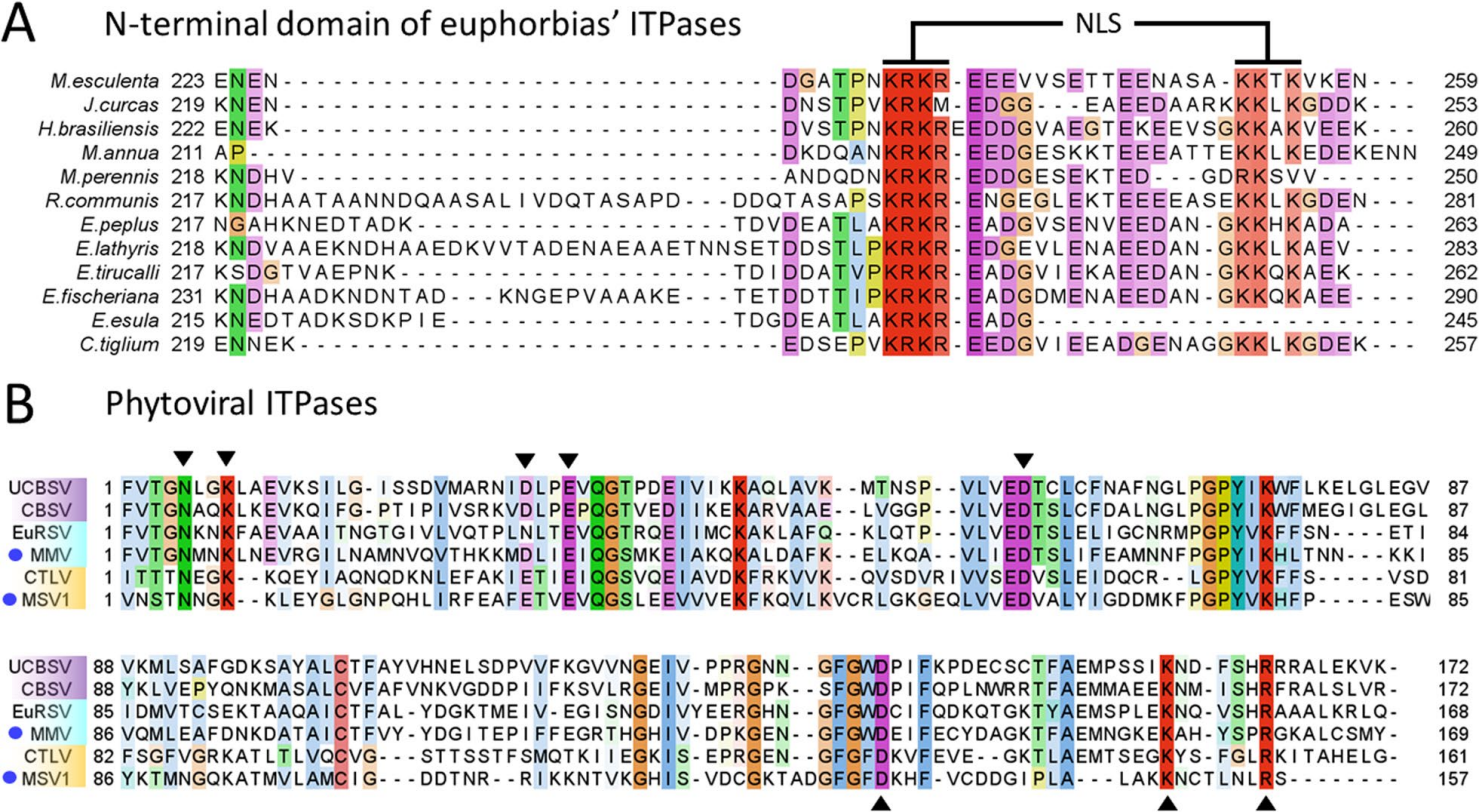
Sequence analysis for the ITPases of the euphorbias and phytoviruses. **A**. Alignment for the C-terminal domain of the euphorbias’ ITPases. Numbers correspond to sequence limits. The conserved motifs of the putative nuclear localization signal (NLS) are highlighted in red.** B**. Alignment of the phytoviral ITPases. Black arrows indicate motifs involved in catalysis and substrate binding. Ipomoviral (purple), potyviral (blue) and secoviral (orange) sequences are grouped together. The new viruses are highlighted by blue circles. For both alignments, conserved residues are highlighted with Clustal colors

The presence of ITPase genes in viruses infecting *M. perennis* advocates that this plant species accumulates high levels of ITP/XTP. It was recently shown that the ITPases encoded by three other euphorbias (i.e. *M. esculenta*, *Jatropha curcas* and *H. brasiliensis*) harbor a C-ter domain not found in other plant ITPases, and containing a predicted nuclear localization signal (NLS) consisting of the basic residues KRKR [19]. This NLS hypothetically ensures low levels of ITP/XTP in the nucleus in order to safeguard the synthesis of DNA and RNA. In line with this idea, an identical motif is found in the endogenous ITPase of *M. perennis* retrieved from the HTS data (acc. numb. OR555821), as well as in eight other euphorbia species retrieved from online databases (Fig. 5A and Additional file 1: Table S7). Interestingly, a second conserved motif is found in C-ter of the domain, corresponding to the motif KKXK in all *Euphorbiaceae* ITPases except in *M. perennis*, where it is represented by the RK motif. The conservation of this second basic motif suggests that the NLS might be bipartite. BlastP searches failed to evidence a similar C-ter domain in the ITPases encoded by other plant families (data not shown).

The ITPases of both MMV and MSV1 appear to be functional as evidenced by a full-length alignment with homologs from the other phytoviruses (Fig. 5B). In particular, the motifs involved in catalysis and substrate binding are conserved [59]. As these enzymes are located next to the replicases, a role similar to that of the ipomoviral ITPases can be hypothesized. In infected plants, UCBSV ITPase has been detected as a free peptide as well as a fusion with the viral replicase Nlb. This fusion may ensure that an active ITPase dimer co-localizes with and protect the viral replicase. The production of both forms of ITPase is due to a weak proteolytic cleavage caused by a T in the last position of the cleavage site VDTQ/T, which is sub-optimally recognized by the protease Nla-Pro [65]. Interestingly, a T is found at a similar position on the putative cleavage site preceding the MMV ITPase (Fig. 2), advocating for a similar production of free and RdRp-associated forms. In the case of MSV1, the ITPase peptide is preceded by the putative cleavage site SVQ/N (Fig. 2), but whether this site is suboptimal requires in vitro analyses.

**Fig. 6:**
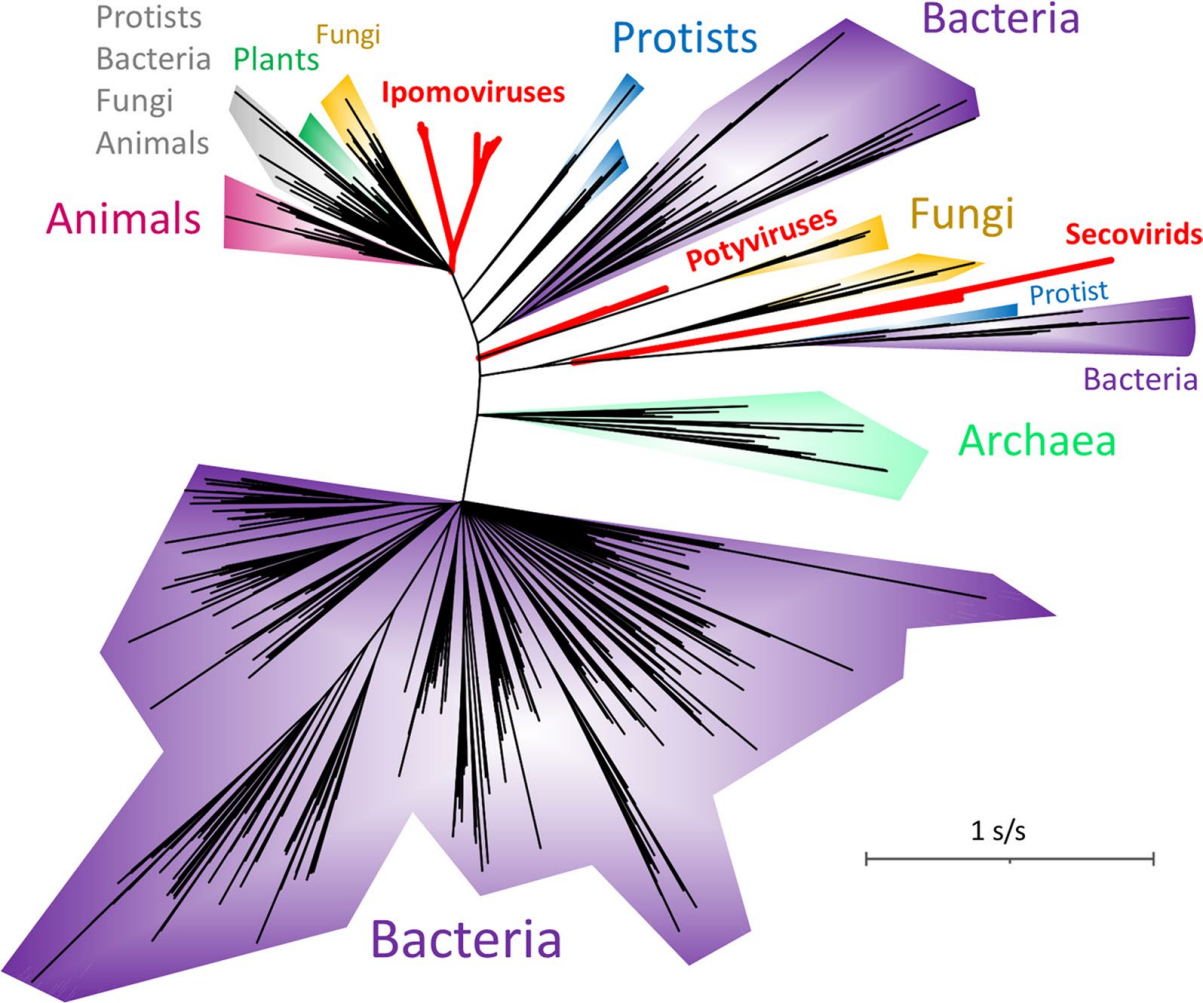
Evolution of the phytoviral ITPases based on an unrooted ML phylogenetic tree. The global tree was built for a curated alignments of representative ITPases using the substitution model LG + F + G4 + I in combination with 1000 bootstrap replicates. Branches with < 80% bootstrap support were collapsed. The tree scale is given in substitution per site. The phytoviral lineages are colored in red

In a global ML phylogenetic tree combining representative sequences of the ITPases encoded by cellular organisms, the phytoviral proteins from the ipomoviruses, potyviruses and secovirids appear on three distant lineages, suggesting three independent HGT events (Fig. 6). The monophyletic nature of the ipomoviral ITPases is supported by their significant homology (> 55% id.). Remarkably, this ipomoviral lineage is close to the ITPases encoded by several groups of eukaryotes, and the best BlastP hits for these viral proteins correspond to euphorbias’ ITPases (Additional file 1: Table S8). The lineage of the potyviral ITPases, which share moderate level of homology (45.2% id.), appears to be related to a clade of fungal enzymes, consistent with BlastP results (Additional file 1: Table S8). Lastly, the monophyletic nature of the secoviral ITPases is less evident as indicated by low homology (40.4% id.), but a common origin is still supported by the fact that CTLV ITPase is yielded as the best hit of a BlastP search on MSV1 protein. For this secoviral lineage, a fungal origin is also possible, yet another origin cannot be excluded considering the long branching in the ML tree and the weak scores of the BlastP results (Additional file 1: Table S8).

## Discussion

Here, HTS analysis of a symptomatic plant of the wild euphorbia *M. perennis* plant led to the identification of three new RNA viruses. In terms of biological characterization, pathogenicity was only demonstrated for MMV, for which the host range seems to be restricted to *Mercurialis* species. It is expected that this virus, like other potyviruses, uses an aphid as vector, but small-scale transmission assays using the common species *M. persicae* and *A. fabae* were unsuccessful. The sole aphid species reported on *M. perennis* is *Aulacorthum solani* [20], and whether this species represents a vector of MMV needs to be tested.

As far as MPV1 and MSV1 are concerned, biological characterization was not possible as mechanical inoculations were ineffective. Nevertheless, a vertically-transmitted asymptomatic infection can be hypothesized for these viruses. First of all, this is supported by the fact that only MMV was sufficient to reproduce the symptoms initially observed on *M. perennis*. In addition, no symptom has been associated with any plant-infecting deltapartitiviruses, which are known to be pollen-transmitted persistent viruses [40, 50], suggesting a similar lifestyle for MPV1. In the case of MSV1 and the other “mercomoviruses”, there are several clues pointing towards a similar lifestyle. Indeed, the related viruses BnSV and PpSV2 were both retrieved from RNAseq data associated with healthy plants [56]. In terms of spread, a vertical transmission is supported for PpSV2 as it was associated with a seed sample. Furthermore, vertical transmission by pollen may explain the identification of “mercomoviral” contigs with high coverages in two pollinator-associated transcriptomes. In fact, numerous other secovirids are known to be pollen-transmitted [61], and a recent large-scale study has shown that partitivirids and secovirids are commonly detected in the pollen of wild plants [13]. It is imaginable that the presence of BnSV isolates with low coverages in vertebrate-associated SRA was caused by contaminating infected pollen, as recently described for Solanum nigrum ilarvirus 1 [47]. Nevertheless, given the low coverage of genes attributed to plant material, contamination during sequencing of multiple simultaneous samples cannot be formally excluded for these two SRAs.

The identification of intact ITPases in the genomes of MMV and MSV1 illustrates the continuous arms race raging between plants and their viruses. Indeed, these enzymes are likely maintained in the phytoviral genomes in order to protect the viral RdRp from the abundant ITP/XTP found in plant tissues, as demonstrated for ipomoviruses infecting cassava [65]. It appears that this presumed defense mechanism against RNA viruses has emerged early in the evolution of euphorbias, since the putative C-ter NLS is conserved among the endogenous ITPases of many distantly-related species of this family but absent in homologs from other plants.

A global phylogenetic analysis suggested that the phytoviral ITPases have been acquired via three distinct HGT events. The ITPases of ipomoviruses may originate from a plant, and in particular from an euphorbia. Hence, it is easily imaginable that this HGT resulted from the RNA-RNA recombination between a precursor ipomoviral RNA and an euphorbia ITPase mRNA. More surprisingly, a fungal origin is suspected for the potyviral enzymes. Last, the secoviral ITPases may originate from a fungus as well, but other groups of cellular organism cannot be excluded. A bacterial origin is possible, similar to the phytoviral *Alkb* and the *Glycosyltransferase 28* domain found on endornaviral polyproteins [58, 66]. How such HGT occurs from a non-plant organism to a phytovirus is unclear. Recent studies have evidenced the transport of mRNA from fungal pathogens to plants [6], and this could hypothetically lead to an RNA-RNA recombination with a replicating potyviral/secoviral virus. In the potyviral genomes, the region corresponding to the ITPase gene insertion corresponds to the Nlb-CP junction that is a known mutational hotspot [41], which may have favored such recombination.

In contrast to MMV and MSV1, no ITPase was detected in the dsRNA genome of MPV1. Likewise, several other ( +)ssRNA viruses have been identified in euphorbias including cassava [5, 24, 48], yet none of these viruses encode for an ITPase. It is possible that the RdRps of these viruses are unaffected by, or less sensitive to ITP/XTP in comparison to the RdRps of poty/seco/ipomoviruses. Furthermore, no ITPase was detected in any euphorbia DNA viruses. In that case, these enzymes are not necessary for DNA viruses replicating inside the nucleus, where they face low levels of ITP/XTP thanks to the endogenous ITPases, but may be required for reverse-transcribing DNA viruses with a cytoplasmic RNA stage.

Last, another specific feature of the phytoviral ITPases is their association with viruses infecting perennial hosts, reflecting the situation of the phytoviral *Alkb* genes [66]. So far, the levels of ITP/XTP have only been examined in cassava, calling for additional investigations on other euphorbias including annual and perennial species.

## Conclusion

Here, three new members of the families *Potyviridae*, *Secoviridae* and *Partitiviridae* infecting *M. perennis* were characterized. The secovirid belongs to a putatively new *Comovirinae* genus named “Mercomovirus”. Strikingly, the potyvirid and secovirid encode for ITPases, which appear to have been acquired multiple times by RNA plant viruses from cellular organisms, most likely from plants and fungi. These enzymes hypothetically protect the viral replicases from the inhibitory concentrations of ITP/XTP found in the cytoplasm of several perennial euphorbias, hence countering a putative plant antiviral defense. Future studies should aim to understand why ITPases are found in some but not all euphorbia RNA viruses. The quantification of ITP/XTP in additional *Euphorbiaceae* species is also needed.

### Supplementary Information

**Additional file 1. Figure S1.** Reads counts (RC) on the viral contigs associated with MMV (blue), MSV1 (orange), MPV1 (green). CP: contig position. **Figure S2.** Uncropped gels used for the Figure 1G. **Figure S3.** ML phylogenetic tree for the CP of MSV1 and related viruses. The tree was built using the substitution model rtREV+F+G4. Black circles on branches indicate >75 % bootstrap support (1000 replicates). The scale is given in substitution per site. Members of the genus *Satsumavirus* were used to root the initial tree. **Table S1.** Primers used in this study. **Table S2.** BlastX report for the viral contigs identified in the HST analysis of *M. perennis*. **Table S3.** Domains and homology identified on the viral ORFs of MMV, MSV1 and MPV1. **Table S4.** Accession numbers for the proteins used for the phylogenetic analyses of the *Potyviridae*. **Table S5.** Accession numbers for the proteins used for the phylogenetic analyses of the *Secoviridae*. **Table S6.** Accession numbers for the RdRp used for the phylogenetic analyses of the *Partitiviridae*. **Table S7.** Accession numbers for the ITPases of the euphorbias. **Table S8.** Most significant (non-viral) BlastP hits for the phytoviral ITPases.

## Data Availability

Not applicable.

## Abbreviations

(+)ssRNA: Positive-sense single-stranded RNA
aa: Amino acid
ACV2: Alloteropsis cryptic virus 2
BnSV: Boehmeria nivea secovirus
CBSV: Cassava brown streak virus
CMV: Celery mosaic virus
CP: Capsid protein
CTAB: Cetyltrimethylammonium bromide
CTLV: Cassava torrado-like virus
CTV1: Carrot torradovirus 1
CVYV: Cucumber vein yellowing virus
dsRNA: Double-stranded RNA
dpi: Days post inoculation
EDTA: Ethylenediaminetetraacetic acid
EuRSV: Euphorbia ringspot virus
FiCV: Fig cryptic virus
HGT: Horizontal gene transfer
HTS: High-throughput sequencing
ICTV: International Committee on Taxonomy of Viruses
ITP: Inosine triphosphate
ITPase: Inosine triphosphate pyrophosphatase
JSV1: Jujube-associated secovirus 1
LINV: Lettuce Italian necrotic virus
LNLCV: Lettuce necrotic leaf curl virus
ML: Maximum-likelihood
MMV: Mercurialis mosaic virus
MYMV: Motherwort yellow mottle virus
MPV1: Mercurialis partitivirus 1
MSV1: Mercurialis secovirus 1
NLS: Nuclear localization signal
NTP: Nucleoside triphosphate
nt: Nucleotide
ORF: Open reading frame
PaCV1: Panax cryptic virus 1
PeCV1: Pepper cryptic virus 1
PIPO: Pretty interesting Potyviridae ORF
PpSV2: Paris polyphylla secovirus 2
PPV: Plum pox virus
Pro: Protease
PVY: Potato virus Y
RCV1: Rose cryptic virus 1
RdRp: RNA-dependent RNA polymerase
RMMV: Ranunculus mild mosaic virus
RsCV3: Raphanus sativus cryptic virus 3
RT-PCR: Reverse transcription-polymerase chain reaction
SBP: Substrate-binding pocket
SMV: Scallion mosaic virus
SRA: Sequence Read Archives
SVYV: Squash vein yellowing virus
TEM: Transmission electron microscopy
TMMV: Tomato mild mottle virus
TuMV: Turnip mosaic virus
UCBSV: Uganda cassava brown streak virus
XTP: Xanthosine triphosphate
