# Supplementary material for: Mixed infection of ITPase-encoding potyvirid and secovirid in Mercurialis perennis: evidences for a convergent euphorbia-specific viral counterstrike
Source: Virol J. 2024 Jan 4;21:6. doi: 10.1186/s12985-023-02257-y (PMC10768138; doi:10.1186/s12985-023-02257-y)
Supplement: Supplementary file 1 — Additional file 1. Figure S1. Reads counts (RC) on the viral contigs associated with MMV (blue), MSV1 (orange), MPV1 (green). CP: contig position. Figure S2. Uncropped gels used for the Figure 1G. Figure S3. ML phylogenetic tree for the CP of MSV1 and related viruses. The tree was built using the substitution model rtREV+F+G4. Black circles on branches indicate >75 % bootstrap support (1000 replicates). The scale is given in substitution per site. Members of the genus Satsumavirus were used to root the initial tree. Table S1. Primers used in this study. Table S2. BlastX report for the viral contigs identified in the HST analysis of M. perennis. Table S3. Domains and homology identified on the viral ORFs of MMV, MSV1 and MPV1. Table S4. Accession numbers for the proteins used for the phylogenetic analyses of the Potyviridae. Table S5. Accession numbers for the proteins used for the phylogenetic analyses of the Secoviridae. Table S6. Accession numbers for the RdRp used for the phylogenetic analyses of the Partitiviridae. Table S7. Accession numbers for the ITPases of the euphorbias. Table S8. Most significant (non-viral) BlastP hits for the phytoviral ITPases. [file 12985_2023_2257_MOESM1_ESM.docx]

**Supplementary figures**

**
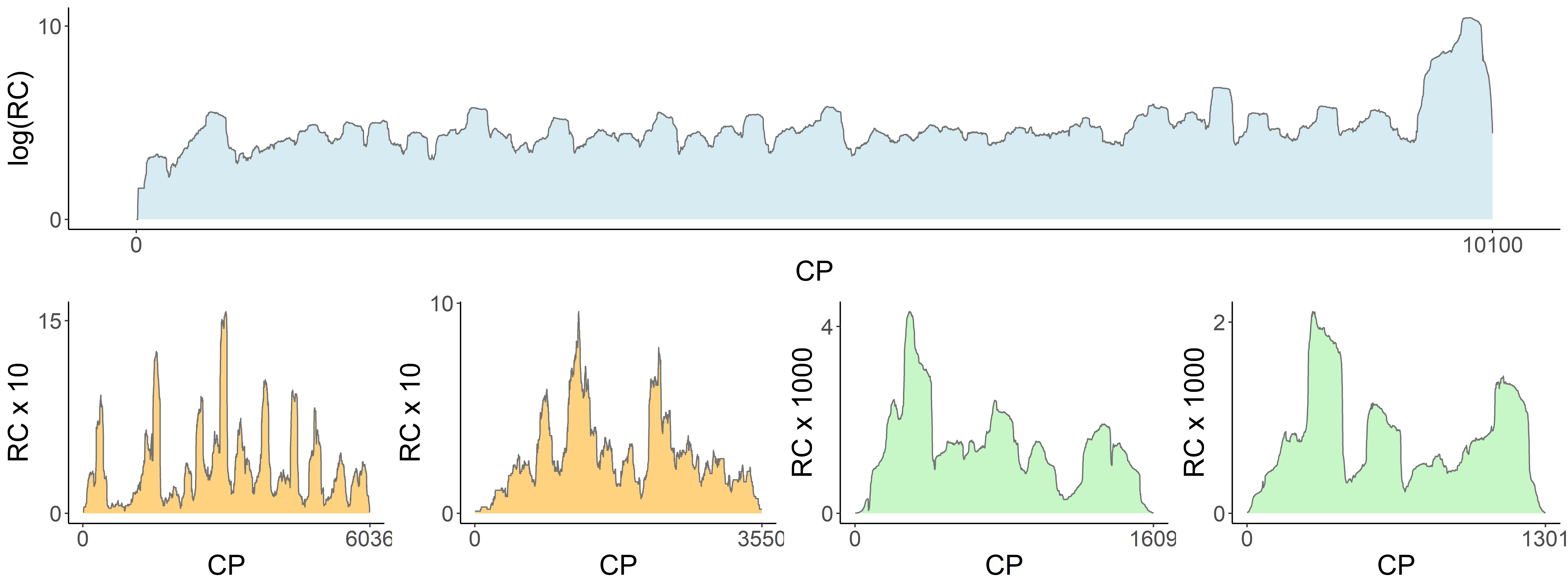
**

**Figure S1.** Reads counts (RC) on the viral contigs associated with MMV (blue), MSV1 (orange), MPV1 (green). CP: contig position.


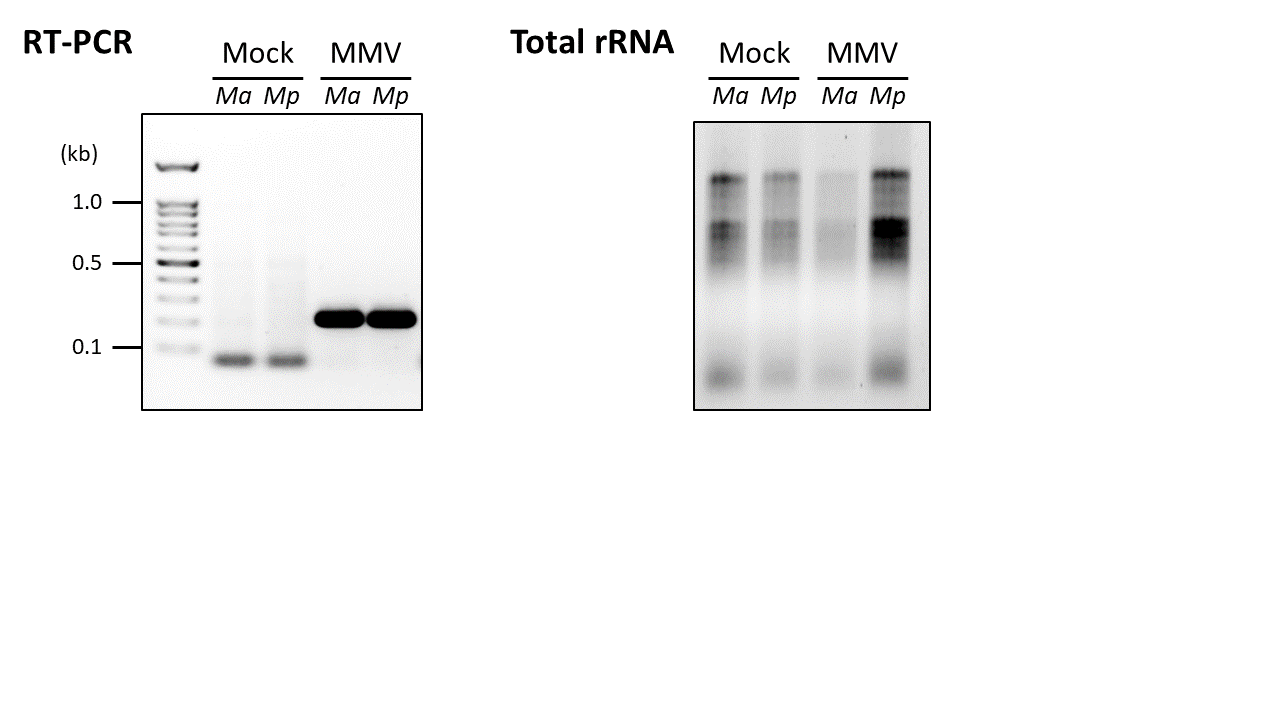


**Figure S2. Uncropped gels used for the Figure 1G.**

**
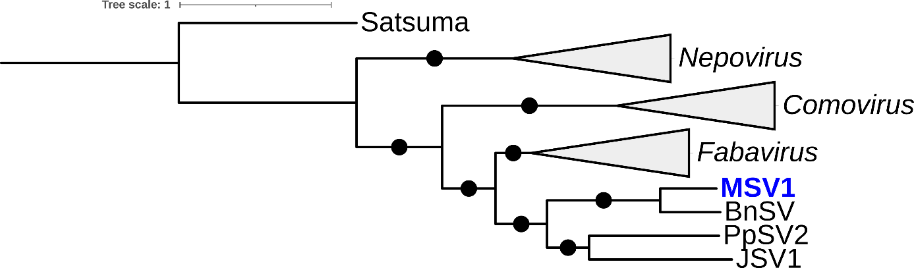
**

**Figure S3. ML phylogenetic tree for the CP of MSV1 and related viruses.** The tree was built using the substitution model rtREV+F+G4. Black circles on branches indicate >75 % bootstrap support (1000 replicates). The scale is given in substitution per site. Members of the genus *Satsumavirus* were used to root the initial tree.

**Supplementary tables**

**Table S1.** Primers used in this study.

| **Name** | **Sequence** | **Use** |
| --- | --- | --- |
| 9721F | GACCCATGATTGAACACGCA | Detection of MMV RNA and 3’ termini |
| 9940R | TGTATGTGTGCTTCTCGGGC |  |
| MercuSeco1F | GAAATAGGCGCCATGTGCTG | Detection of MSV1 RNA1 |
| MercuSeco1R | TGCACCACTCGTTCCATACC |  |
| MercuSeco2F | TATGACCACGCGAAGAGGTG | Detection of MSV1 RNA2 |
| MercuSeco2R | CCACTGCATTATTGCTCCGC |  |
| MercuPartiti1F | TGAGGGCCCATGACGTACTA | Detection of MPV1 RNA1 |
| MercuPartiti1R | GGCGCAGCGACTTTCTTATG |  |
| MercuPartiti2F | CGAGAGGACGCACCTATTCC | Detection of MPV1 RNA2 |
| MercuPartiti2R | GAGCGTCAAATTTCGGGTCG |  |
| SecoRN1ter | TCCCTATGGTCTTGGGAGAGG | 3’ termini of MSV1 RNA1 |
| SecoRN2ter | TGGTCCAGAAGGGAGTAGTGG | 3’ termini of MSV1 RNA2 |
| PartitiRN1ter | AGTCGGAGACGATAGCTTGA | 3’ termini of MPV1 RNA1 |
| PartitiRN2ter | TCGCTCCAATCCACGAACAA | 3’ termini of MPV1 RNA2 |
| Racepotytwo | CCACCTGCTCGGATGGACCCTCA | 5’ termini of MMV |
| SecoRNA15one | CCCTGCGCCTCCGCAGATAATTCTTCAT | 5’ termini MSV1 RNA1 |
| SecoRNA25one | CCTGCGGGCGCACTTGAGTTCTAAAAGG | 5’ termini of MSV1 RNA2 |
| PartitiRN1t5one | TGCGACTTCACGTCTCACAA | 5’ termini of MPV1 RNA1 |

**Table S2.** BlastX report for the viral contigs identified in the HST analysis of *M. perennis*.

| **Contig** | **Length (bp)** | **Best BlastX hit** | **Accession** | **Cover. (%)** | **Id. (%)** |
| --- | --- | --- | --- | --- | --- |
| 1 | 10,100 | Euphorbia ringspot virus polyprotein | YP_009305422.1 | 89 | 53 |
| 2 | 6,036 | Boehmeria nivea secovirus polyprotein 1 | DAZ91064.1 | 89 | 67 |
| 3 | 3,550 | Boehmeria nivea secovirus polyprotein 2 | DAZ91065.1 | 83 | 59 |
| 4 | 1,609 | Raphanus sativus cryptic virus 3 RdRp | YP_002364401.1 | 93 | 81 |
| 5 | 1,305 | Raphanus sativus cryptic virus 3 CP | YP_002364402.1 | 81 | 59 |

**Table S3.** Domains and homology identified on the viral ORFs of MMV, MSV1 and MPV1.

| **Pfam** | **Positions (aa)** | **E-value** | **Annotation** |
| --- | --- | --- | --- |
| **MMV polyprotein** | | | |
| PF01577 | 197-360 | 8.4e-27 | Potyvirus P1 protease (P1-Pro) |
| PF00851 | 385-819 | 2e-122 | Helper component proteinase (HC-Pro) |
| PF13608 | 835-1274 | 2.5e-73 | Protein P3 of Potyviral polyprotein (P3) |
| PF00270 | 1300-1434 | 5.9e-06 | DEAD/DEAH box helicase |
| PF00271 | 1472-1582 | 9.6e-10 | Helicase conserved C-terminal domain |
| PF08440 | 1608-1881 | 4e-70 | Potyviridae polyprotein |
| PF00863 | 2101-2333 | 2.4e-57 | Peptidase family C4 (Nla-Pro) |
| PF00680 | 2384-2802 | 1e-77 | RdRp (NlB) |
| PF01725 | 2855-3024 | 1.7e-46 | Ham1 family (Ham1) |
| PF00767 | 3074-3291 | 3.7e-79 | Potyvirus coat protein (CP) |
| **MSV1 RNA1 polyprotein** | | | |
| - | 1-477 | - | Homology to fabaviruses protease cofactor (Col-Pro) |
| PF00910 | 477-582 | 4.7e-23 | RNA helicase (Hel) |
| PF00548 | 1114-1151 | 2.2e-4 | 3C cysteine protease (Pro) |
| PF00680 | 1223-1684 | 4.3e-77 | RdRp (Pol) |
| PF01725 | 1898-2034 | 2e-09 | Ham1 family (Ham1) |
| **MSV1 RNA2 polyprotein** | | | |
| - | 1-415 | - | Homology to fabavirus movement protein (MP) |
| PF02247 | 415-762 | 3.9e-37 | Comoviridae large coat protein (LCP) |
| PF02248 | 838-973 | 9.3e-07 | Comoviridae small coat protein (SCP) |
| **MPV1 RNA1 protein** | | | |
| PF00680 | 41-458 | 5.7e-92 | RdRp |
| **MPV1 RNA2 protein** | | | |
| - | - | - | Homology to putative partitivirus coat protein (CP) |

**Table S4.** Accession numbers for the proteins used for the phylogenetic analyses of the *Potyviridae*.

| **Genus** | **Virus** | **Polyprotein** |
| --- | --- | --- |
| ***Potyvirus*** | Euphorbia ringspot virus (EuRSV) | YP 009305422.1 |
|  | Turnip mosaic virus (TuMV) | BAQ56293.1 |
|  | Plum pox virus (PPV) | AWJ58379.1 |
|  | Celery mosaic virus (CMV) | YP 004376199.1 |
|  | Lettuce Italian necrotic virus (LINV) | ARF07716.1 |
|  | Ranunculus mild mosaic virus (RMMV) | BCS79971.1 |
|  | Scallion mosaic virus (SMV) | NP 570725.1 |
| ***Arepavirus*** | Areca palm necrotic ringspot virus | YP_010087001.1 |
|  | Areca palm necrotic spindle-spot virus | YP_009553653.1 |
| ***Celavirus*** | Celery latent virus | YP_010087166.1 |
| ***Tritimovirus*** | Wheat streak mosaic virus | NP_046741.1 |
|  | Brome streak mosaic virus | NP_612585.1 |
| ***Roymovirus*** | Rose yellow mosaic virus | YP_006905847.1 |
|  | Passiflora edulis symptomless virus | UIX56007.1 |
| ***Poacevirus*** | Triticum mosaic virus | YP_002956073.1 |
|  | Sugarcane streak mosaic virus | ALB39049.1 |
| ***Macluravirus*** | Artichoke latent virus | YP_009129267.1 |
|  | Yam chlorotic necrotic mosaic virus | YP_009507672.1 |
| ***Bymovirus*** | Barley yellow mosaic virus | NP_148999.1 |
|  | Oat mosaic virus | NP_659025.1 |
| ***Brambyvirus*** | Blackberry virus Y | YP_851006.1 |
| ***Ipomovirus*** | Cassava brown streak virus (CBSV) | YP_007027011.1 |
|  | Ugandan cassava brown streak virus (UCBSV) | ANI24981.1 |
|  | Cucumber vein yellowing virus (CVYV) | QGW63304.1 |
|  | Squash vein yellowing virus (SVYV) | AEV45694.1 |
|  | Tomato mild mottle virus (TMMV) | QWT83742.1 |
| ***Bevemovirus*** | Bellflower veinal mottle virus | YP_009508455.1 |
| ***Rymovirus*** | Japanese yam mosaic virus | AJD23395.1 |
|  | Agropyron mosaic virus | YP 025106.1 |
|  | Hordeum mosaic virus | YP 025107.1 |

**Table S5.** Accession numbers for the proteins used for the phylogenetic analyses of the *Secoviridae*.

| **Genus** | **Virus name** | **RNA1 polyprot.** | **RNA2 polyprot** |
| --- | --- | --- | --- |
| **«Mercomovirus»** | Jujube-associated secovirus 1 (JSV1) | QNN26328.1 | QNN26327.1 |
|  | Boehmeria nivea secovirus (BnSV) | DAZ91064.1 | DAZ91065.1 |
|  | Paris polyphylla secovirus 2 (PpSV2) | DAZ91073.1 | DAZ91072.1 |
| ***Sadwavirus*** | Citrus mosaic sadwavirus | BAH56590.1 | BAA92934.1 |
|  | Satsuma dwarf virus | QPP19762.1 | Q9WAL9.1 |
| ***Cheravirus*** | Alpine wild prunus virus | UZP65117.1 | UZP65118.1 |
|  | Cherry rasp leaf virus | ANC51274.1 | YP_081445.1 |
|  | Arracacha virus B | QCY50805.1 | QCY50806.1 |
|  | Apple latent spherical virus | NP_620568.1 | NP_620569.1 |
| ***Torradovirus*** | Carrot torradovirus 1 (CTV1) | BBH72270.1 | BBH72272.1 |
|  | Motherwort yellow mottle virus (MYMV) | YP_009389541.1 | YP_009389545.1 |
|  | Lettuce necrotic leaf curl virus (LNLCV) | QRN45896.1 | YP_009389543.1 |
|  | Cassava Torrado-like virus (CTLV) | AWW14943.1 | UZN89640.1 |
| ***Waikavirus*** | Maize chlorotic dwarf virus | QHB15177.1 | - |
|  | Bellflower vein chlorosis virus | YP_009165498.1 | - |
|  | Maize chlorotic dwarf virus | QAT96400.1 | - |
| ***Sequivirus*** | Lettuce star mosaic virus | QSM07372.1 | - |
|  | Carrot necrotic dieback virus | UBX89813.1 | - |
|  | Parsnip yellow fleck virus | NP_619734.1 | - |
| ***Fabavirus*** | Prunus virus F | ANH71251.1 | UBZ25945.1 |
|  | Cherry virus F | AZZ10050.1 | UBZ25940.1 |
|  | Peach leaf pitting-associated virus | ATD53314.1 | QED43052.1 |
|  | Broad bean wilt virus 2 | AGM38171.1 | WCD56771.1 |
| ***Comovirus*** | Cowpea mosaic virus | QWT83580.1 | P03599.1 |
|  | Red clover mottle virus | NP_620468.1 | AWK57383.1 |
|  | Turnip ringspot virus | ADZ54704.1 | ACX53647.1 |
|  | Phaseolus vulgaris severe mosaic virus | QJC19295.1 | QJC19296.1 |
|  | Bean pod mottle virus | ADD92352.1 1 | AAW69771.1 |
|  | Arabidopsis latent virus-1 | QCE32095.1 | QCE32096.1 |
| ***Nepovirus*** | Grapevine fanleaf virus | WBR64808.1 | APD26307.1 |
|  | Artichoke yellow ringspot virus | YP_009508092.1 | (only partial) |
|  | Petunia chlorotic mottle virus | YP_009342468.1 | YP_009342469.1 |
|  | Melon mild mottle virus | YP_009507921.1 | YP_009507922.1 |
|  | Tomato ringspot virus | NP_620765.1 | NP_620762.1 |
| ***Dicistrovirus*** | Cricket paralysis virus | BCZ95743.1 | - |
|  | Drosophila C virus | QEQ50987.1 | - |

**Table S6.** Accession numbers for the RdRp used for the phylogenetic analyses of the *Partitiviridae*.

| **Genus** | **Virus** | **RdRp** |
| --- | --- | --- |
| ***Gammapartitivirus*** | Fusarium solani virus 1 | NP_624350.1 |
|  | Penicillium stoloniferum virus S | YP_052856.2 |
|  | Magnaporthe oryzae partitivirus 1 | APP18151.1 |
|  | Pythium nunn virus 1 | YP_009551507.1 |
| ***Deltapartitivirus*** | Fig cryptic virus (FiCV) | YP_004429258.1 |
|  | Rose cryptic virus 1 (RCV1) | ABZ10945.1 |
|  | Raphanus sativus cryptic virus 3 (RsCV3) | YP_002364401.1 |
|  | Alloteropsis cryptic virus 2 (ACV2) | QRG29179.1 |
|  | Panax cryptic virus 1 (PaCV1) | QED42879.1 |
|  | Pepper cryptic virus 1 (PeCV1) | AVV48358.1 |
| ***Cryspovirus*** | Cryptosporidium parvum virus 1 | AAC47805.1 |
| ***Betapartitivirus*** | Atkinsonella hypoxylon virus | NP_604475.1 |
|  | Rosellinia necatrix partitivirus 1-W8 | BAD98237.1 |
| ***Alphapartitivirus*** | Heterobasidion partitivirus 3 | ACO37245.1 |
|  | White clover cryptic virus 1 | YP_086754.1 |
| ***Picobirnavirus*** | Human picobirnavirus | YP_239361.1 |
|  | Otarine picobirnavirus | AFJ79071.1 |

**Table S7.** Accession numbers for the ITPases of the euphorbias.

| **Species** | **Accession number** |
| --- | --- |
| *Manihot esculenta* | XP_021606115.1 |
| *Jatropha curcas* | XP_012077670.1 |
| *Hevea brasiliensis* | XP_021636042.1 |
| *Mercurialis annua* | XP_050219982.1 |
| *Ricinus communis* | XP_015574043.3 |
| *Euphorbia peplus* | WCJ24204.1 |
| *Euphorbia lathyris* | GGMH01006714.1 |
| *Euphorbia tirucalli* | GETW01020935.1 |
| *Euphorbia fischeriana* | JL054653.1 |
| *Euphorbia esula* | DV126422.1 |
| *Croton tiglium* | GGDV01044231.1 |

**Table S8.** Most significant (non-viral) BlastP hits for the phytoviral ITPases

| **ITPase** | **Best BlastP hit** | **Species** | **Cover. (%)** | **E-value** | **Id. (%)** |
| --- | --- | --- | --- | --- | --- |
| CBSV | XP_015574043.3 | *Ricinus communis* (plant) | 100 | 2e-70 | 61.63 |
| UCBSV | WCJ24204.1 | *Euphorbia peplus* (plant) | 100 | 2e-68 | 60.47 |
| EuRSV | CAI5759911.1 | *Candida verbasci* (yeast) | 100 | 9e-39 | 43.65 |
| MMV | WFD47328.1 | *Malassezia furfur* (ascomycete) | 91 | 1e-45 | 50.00 |
| MSV1 | KAI6171434.1 | *Aphelenchoides bicaudatus* (nematod) | 85 | 4e-06 | 31.08 |
| CTLV | XP_040650895.1 | *Penicillium griseofulvum* (ascomycete) | 93 | 3e-16 | 37.04 |
